# The Hybrid Position/Force Walking Robot Control Using Extenics Theory and Neutrosophic Logic Decision

**DOI:** 10.3390/s22103663

**Published:** 2022-05-11

**Authors:** Ionel-Alexandru Gal, Alexandra-Cătălina Ciocîrlan, Luige Vlădăreanu

**Affiliations:** Institute of Solid Mechanics of the Romanian Academy, 15 C. Mille, 010141 Bucharest, Romania; alexandru.gal@imsar.ro (I.-A.G.); alexandra.ciocirlan@imsar.ro (A.-C.C.)

**Keywords:** hybrid position/force control, sliding mode control, decision method, neutrosophic logic, extension set

## Abstract

This paper presents a hybrid force/position control. We developed it for a hexapod walking robot that combines multiple bipedal robots to increase its load. The control method integrated Extenics theory with neutrosophic logic to obtain a two-stage decision-making algorithm. The first stage was an offline qualitative decision-applying Extenics theory, and the second was a real-time decision process using neutrosophic logic and DSmT theory. The two-stage algorithm separated the control phases into a kinematic control method that used a PID regulator and a dynamic control method developed with the help of sliding mode control (SMC). By integrating both control methods separated by a dynamic switching algorithm, we obtained a hybrid force/position control that took advantage of both kinematic and dynamic control properties to drive a mobile walking robot. The experimental and predicted results were in good agreement. They indicated that the proposed hybrid control is efficient in using the two-stage decision algorithm to drive the hexapod robot motors using kinematic and dynamic control methods. The experiment presents the robot’s foot positioning error while walking. The results show how the switching method alters the system precision during the pendulum phase compared to the weight support phase, which can better compensate for the robot’s dynamic parameters. The proposed switching algorithm directly influences the overall control precision, while we aimed to obtain a fast switch with a lower impact on the control parameters. The results show the error on all axes and break it down into walking stages to better understand the control behavior and precision.

## 1. Introduction

Worldwide, practical robot applications are diversifying more and more in the new world of robotics, automation, and artificial intelligence [1]. Researchers and engineers are working on developing solutions and solving problems for all kinds of robots. This research will enhance human motion and workspace investigation through different sensors and automate tasks for unattended robots [2]. Into this category falls every type of robot control method that can improve robot control and behavior, with or without autonomous capabilities [3]. For a robot to be capable of accomplishing a designated task, it must reject most of the uncertainties and disturbances within the work environment. The robot must also handle information from several sensors and fuse the data to reach a close decision to the truth value.

Most mobile robots combine kinematic and dynamic control methods to solve such a problem, each designated for certain joints in the robot structure. However, for a highly versatile robot structure, a hybrid position/force method is used. Although the technique is not new, having begun with Raibert and Craig [4], it has the attention of robotic control research, and it continues to bring adaptability to the robots using it. In recent years, different approaches have been researched. Zhang et al. [5] created a hybrid control method that could adjust the joint dynamic parameters online. The process allowed rough modeling of the robot parameters and left the fine-tuning to the control algorithm. A similar approach used a neural network to autocalibrate the control parameters [6]. Still, not all hybrid methods can use mechanical parameters because they increase the system’s complexity. For a robot with a simple structure for which the kinematic or dynamic equations are easier to define, a classic approach to hybrid control can be easier to implement. Some examples include the hybrid method with impedance control [7] or even the backstepping method with a Hamilton controller [8]. Applications of the hybrid control method can be found in all types of robots, from a robot used for mechanical tests [9] to an upper limb rehabilitation robot [10]. Because of its versatility, the hybrid position/force control was chosen as the primary control method for the walking robot.

As we know, hybrid control combines a kinematic control method for joints that do not require compensation of weight and inertia and a dynamic control method that can handle these parameters and reject environmental disturbances. The kinematic approach is used for positioning control of the robot and the dynamic approach for the force and torque control. Consequently, a classic proportional-integrative-derivative (PID) regulator was chosen for the position control and a sliding mode control (SMC) method [11] for the force and torque control. The main reason for using a SMC method was its robustness in the presence of external disturbances and uncertainties. Many scientists have used the control method to improve industrial robot trajectory [12], mobile robot trajectory in dynamic environments [13], n-link serial manipulator control [14], balance control of a two-wheel robot [15], and even airplane fuselage inspection [16]. SMC is not a perfect control method, and it has drawbacks, one of which is the chattering effect that it can introduce. New research is published every year [17] on eliminating the chattering impact in a general manner or for a specific robot structure or purpose.

Using multiple regulators or control methods on the same robot structure can separate the robot joints statically into two categories, starting with the design of the control law. However, this is not desirable if one needs to build a versatile robot. Hence, a real-time decision method must determine the degree of freedom (joints) controlled by each method. A combination of techniques and control methods was thus selected. The first one, Extenics [18] or extension logic [19], entails defining the control parameters and robot properties or abilities and is used by scientists to configure problem-solving algorithms [18] and even design toys for children with special needs [20].

For the proposed robot structure, Extenics helped ease the process of organizing the parameters of each control method and provided the offline means of solving potential conflicts, uncertainties, or mismatching of sensor data and regulators.

Neural networks [21,22] were considered for the decision method, but the process of training the network was too extensive for the proposed robot. Another possibility was using swarm optimization [23] to predict what the robot needs in terms of control methods. This also overcomplicated the control system, and it should be handled in future work. For the presented robot, neutrosophic logic [24] and the Dezert–Smarandache Theory (DSmT) [25] were chosen. DSmT combined with Extenics is used to manage decision making [20] and provides excellent results by combining the mapping process of Extension logic with the sensor fusion of DSmT in uncertain and contradictory conditions. As an extension of fuzzy logic, neutrosophic logic and DSmT have been used by researchers to develop applications [26] for aviation parking [27], multi-UAV surveillance [28], obstacle avoidance in unknown environments [29], and environmental detection and estimation [30].

A different approach in designing the decision algorithm of a hybrid system is using time triggers or an event-driven mechanism [31] with an event generation mechanism [32] to ensure control of the system at the precise times of important defined events. This approach is safe for robots in a known environment, but it can fail or make inconsistent decisions for robots moving inside unknown and unstructured environments.

To develop a mobile walking robot, one can reference many highly advanced robots, some designed by renowned institutes [33], that use dynamic control methods to provide stability and error rejection for the control architecture. While efficient mathematical solutions [34] are desired for a control law to give it low computational requirements, these can be difficult to obtain when the robot model is complex. Moreover, the dynamic control of any robot must overcome external disturbances [35] and reject any influence from other sources, including within the sensor information.

Here, we propose and describe a mobile walking robot hybrid position/force control that can be used within a group of linked robots. The research aim to obtain a control algorithm and method using both kinematic and dynamic control methods and an intelligent switching method between them. As a result, several experiments were conducted to improve the performance of the developed hybrid control, taking advantage of the extension set and neutrosophic logic. The extension set and neutrosophic logic were used to enhance the decision making required by the hybrid control, the first as an offline set of characteristics to extend the system’s definition, and the second as an online switching mechanism that works with uncertain and contradictory information. The resulting hybrid control using a two-stage decision algorithm was a robot control method that took advantage of the best properties of the kinematic and dynamic control laws while the robot was fulfilling its tasks in uncertain environments. The data fusion provided by the neutrosophic theory in contradictory or uncertain conditions improved the decision switching mechanism, while the overall reference tracking of the robot did not decrease. The computational requirements of the proposed hybrid control were reduced because of the kinematic control method when the robot did not require dynamic compensation.

The paper is divided into six main sections. Section 2 provides a visual description of the robot used in the experiments. Section 3 presents the offline decision using the extension set, while Section 4 presents the decision method based on neutrosophic logic that takes advantage of DSmT [25]. The hybrid control is presented in Section 5 with an in-depth analysis of the kinematic (Section 5.1) and SMC dynamic (Section 5.2) control methods. Section 6 and Section 7 contain the conducted experiments and simulations with the obtained results. In the end, Section 8 presents this paper’s conclusions.

## 2. System Description

**Figure 1:**
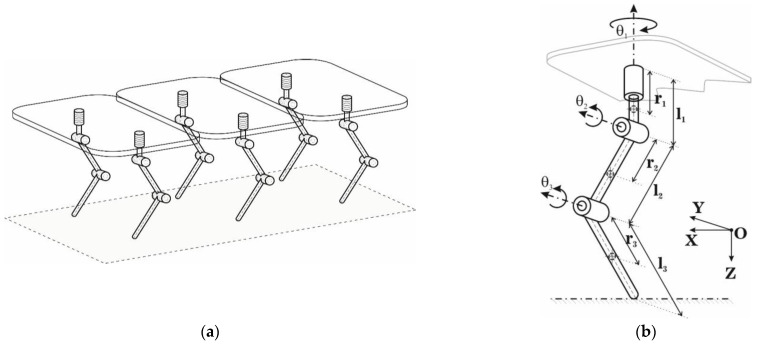
The robot structure: (**a**) the hexapod walking robot; (**b**) kinematic structure of the robot leg.

Figure 1 presents the robot structure. The robot was a hexapod [36], and its design was selected to avoid stability problems. Future research will consider the stability of a single bipedal mobile walking robot. As can be seen, the robot platform was divided into three modules, resulting in a modular robot that could be further extended or reconfigured. Figure 1b presents the kinematic structure of the hexapod robot leg. Each leg had three degrees of freedom, ensuring the 3D positioning of the foot.

In the robot structure, aluminum was considered for rectangular bars with height, width, and length dimensions *h_i_* × *w_i_* × *l_i_* and weight *m_i_*. *h_i_* and *w_i_* with the dimensions of *h_i_* = 3 cm and *w_i_* = 3 cm. Table 1 presents the robot dimensions used in the testing and simulations presented throughout the paper.

## 3. Extenics Theory and Extension Set Applied to Robots

Extenics is a scientific field that uses modeling and formal methods to extend elements or physical objects. The models and methods are then used to solve contradictory problems that cannot be solved in their defined form and conditions [37,38].

Because contradictory problems are omnipresent in any field, Extenics aims to define a set of methods that allows solving contradictory issues using virtual simulation with the help of computers.

The central parts of extension theory are the base element theory, extension set theory, and extension logic [39].

The fundamental element used to describe objects in extension theory is defined as:

(1) $$M=\left(O_{m},c_{m},v_{m}\right)$$

where *M* is the object element for which *O_m_* is the object, *c_m_* is the characteristic, and *v_m_* is the measure. If one characteristic exists, the other matter element can be of only one dimension. If the object has more characteristics, however, the multidimension matter element is be defined as:

(2) $$M=\left[\begin{array}{ccc} O_{m}, & c_{m1}, & v_{m1}\\ & c_{m2}, & v_{m2}\\ & \vdots & \vdots \\ & c_{mn}, & v_{mn} \end{array}\right]=\left(O_{m},C_{m},V_{m}\right).$$

If a hybrid control is used, then a multidimension matter element is used, described by many characteristics specific to the chosen control methods.

For a hybrid position/force control, the following matter element can be defined:

(3) $$R_{0}=\left[\begin{array}{ccc} \mathrm{Robot}\;\mathrm{Control} & \mathrm{Control}\;\mathrm{Type} & \mathrm{Hybrid}\;\mathrm{Control}\\ & \mathrm{Overall}\;\mathrm{Computation}\;\mathrm{Speed} & \mathrm{Good}\\ & \mathrm{Refference}\;\mathrm{Tracking}\;\mathrm{Speed} & \mathrm{Very}\;\mathrm{Good}\\ & \mathrm{Refference}\;\mathrm{Tracking}\;\mathrm{Error} & \mathrm{Very}\;\mathrm{Good}\\ & \mathrm{Inertia}\;\mathrm{Compensation} & \mathrm{Good}\\ & \mathrm{Disturbance}\;\mathrm{Rejection} & \mathrm{Good} \end{array}\right].$$

By using Extenics principle 2.2 [40], which Ren et al. [18] used to design low carbon products, the object from Equation (3) can be extended and decomposed into two matter elements for which *O*_1_ and *O*_2_ are defined as kinematic control and dynamic control, respectively. The two objects have the same characteristics as the primary object (called “hybrid control”) but with different values:

(4) $$R_{1}=\left[\begin{array}{ccc} \mathrm{Robot}\;\mathrm{Control}, & \mathrm{Control}\;\mathrm{Type} & \left\{\begin{array}{c} \mathrm{Kinematic}\;\mathrm{Control}\\ \mathrm{PID}\;\mathrm{Control} \end{array}\right\}\\ & \mathrm{Overall}\;\mathrm{Computation}\;\mathrm{Speed}, & \mathrm{Very}\;\mathrm{Good}\\ & \mathrm{Refference}\;\mathrm{Tracking}\;\mathrm{Speed}, & \mathrm{Very}\;\mathrm{Good}\\ & \mathrm{Refference}\;\mathrm{Tracking}\;\mathrm{Error}, & \mathrm{Good}\\ & \mathrm{Inertia}\;\mathrm{Compensation}, & \mathrm{Very}\;\mathrm{Poor}\\ & \mathrm{Perturbance}\;\mathrm{Rejection} & \mathrm{Poor} \end{array}\right],$$

(5) $$R_{2}=\left[\begin{array}{ccc} \mathrm{Robot}\;\mathrm{Control}, & \mathrm{Control}\;\mathrm{Type} & \left\{\begin{array}{c} \mathrm{Dynamic}\;\mathrm{Control}\\ \mathrm{PID}\;\mathrm{Sliding}\;\mathrm{Control} \end{array}\right\}\\ & \mathrm{Overall}\;\mathrm{Computation}\;\mathrm{Speed}, & \mathrm{Average}\\ & \mathrm{Refference}\;\mathrm{Tracking}\;\mathrm{Speed}, & \mathrm{Good}\\ & \mathrm{Refference}\;\mathrm{Tracking}\;\mathrm{Error}, & \mathrm{Very}\;\mathrm{Good}\\ & \mathrm{Inertia}\;\mathrm{Compensation}, & \mathrm{Very}\;\mathrm{Good}\\ & \mathrm{Perturbance}\;\mathrm{Rejection} & \mathrm{Very}\;\mathrm{Good} \end{array}\right].$$

As can be seen, the two matter elements from Equations (4) and (5) describe the control types, the kinematic and the dynamic, briefly. The matter elements are customized for control types, but numerous other features can be added for which the matter characteristics are different according to the desired control type.

The contradiction between the two control types is found using the matter element characterization. The kinematic type has a better computational speed for a real-time controller. Still, when a robot is subject to inertial forces, it has worse positioning error and tracking speed. On the other hand, the dynamic control method takes into consideration the inertial forces that act on a robot and has a better tracking error. However, although the tracking error is better, the tracking speed is worse. The overall computational speed is greatly diminished, owing to the many calculations inside the control loop.

**Figure 2:**
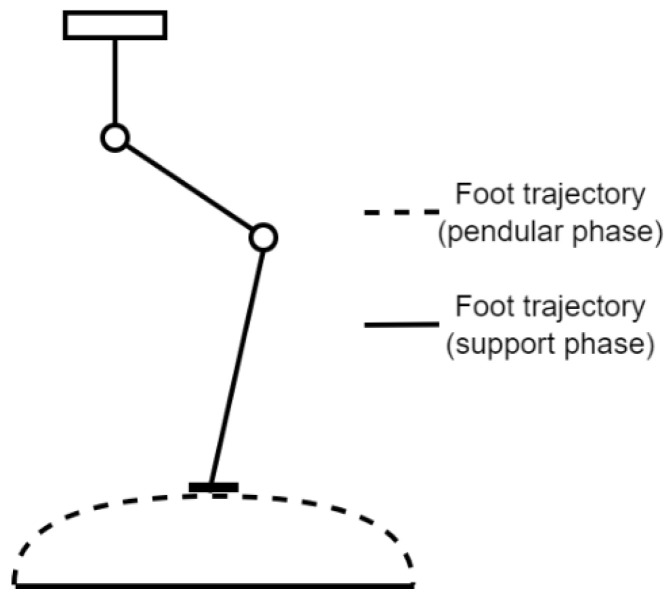
A simple trajectory for the robot foot.

As a simple reference trajectory, an ideal trajectory of the foot (Figure 2) was used. When a robot foot has the role of support (the unbroken line in Figure 2), precise control is needed that considers the weight and inertia of the robot, so the robot’s position does not oscillate on the vertical axis during the support phase. Additionally, the joints of a robot leg must complete or partially support the overall robot weight, including the other legs in the advancing stage. The dotted line in Figure 2 represents the leg balance trajectory when a robot takes a step for which the positioning is not required to be precise but must be fast and smooth. During the second motion phase, the leg joints support only their leg weight.

Knowing the gross characterization of the two types of trajectories that a robot foot takes, the problem of choosing the control type is solved by checking the properties of the two matter elements defined by Equations (4) and (5).

Thereby, to control the robot during the uniform pull and weight support phase, the matter element R_2_ was chosen. Its properties provided better precision in tracking the position reference and considered the robot’s inertia to compensate for the robot’s weight and inertial motion forces.

When the robot foot must follow a curve in space during the advancing phase, the robot’s weight was not supported by it, so we used matter element R_1_. The R_1_ properties better corresponded to the robot motion criteria. The kinematic controller was used when the robot needed to make a forward or reverse motion that was not in direct contact with the support plane. High positioning precision was not required for the advancing movement, only a faster speed to position the foot as quickly as possible on the next support point.

One of the properties of the two matter elements is “overall computation speed,” which indicates how many mathematical operations are needed to compute the actual reference for each separate joint. Therefore, a kinematic controller is much more efficient in computational requirements, performing fewer mathematical operations than a dynamic one. Results have indicated that a kinematic controller supplies not only a better tracking speed but also minimal resource consumption.

When defining and separating the control type that is best for the job, a real-time switching method was required. Thus, with the help of neutrosophic logic, the robot leg phase was determined, based not on reference values but on sensor information. According to the data calculated by Extenics theory on which type of control law to use, a hybrid control could be obtained to resolve the transition problem between kinematic and dynamic control laws. As an offline result, it could be reiterated for future datasets to enhance control properties or add a second layer of details and properties.

## 4. Neutrosophic Logic in Robot Control

As defined in [41], neutrosophic logic is the foundation of neutrosophic mathematics. Neutrosophic logic works with neutrosophic sets that generalize fuzzy sets and describe neutrosophic elements. The elements are based on <A>, <anti A>, and <neutral A>, where <A> is an attribute, <anti A> is the opposite of the attribute, and <neutral A> is the neutral area between <A> and <anti A>.

In neutrosophic logic, every affirmation *Af* is *T%* true, *I%* undetermined (uncertain), or *F%* false. Therefore, we can say *Af* (*T, I, F*)*,* where *T, I*, and *F* are standard or non-standard subsets of the interval ${]}^{-}0,1^{+}[$ [41].

If *U* is the work universe and *M* is a set included in *U*, then one element *x* from *U* is written as *x* (*T*, *I*, *F*) according to set *M* and belongs to the same set in the following way: element *x* is *t%* true in set *M*; element *x* is *i%* undetermined in set *M* (either true or false); and element *x* is *f%* false in set *M*. The value of *t* varies in *T*, *i* varies in *I,* and *f* varies in *F* [42,43].

As described in the current paper, the robot control diagram presented used both kinematic and dynamic elements. At a specific time, the robot used only one of the control methods to maximize and optimize computing and motion speed or the positioning error. A precision element was needed to switch between the two control types. Using Extenics and extension theory, the contradictory elements were defined to separate the two control types between which the decision algorithm switched using neutrosophic theory.

The classic neutrosophic theory [25] chooses between the two control methods. The general equation is presented in Relation (6) and defines the generalized basic belief assignment:

(6) $$m\left(C\right)=\sum_{\begin{array}{c} A,B\in D^{\Theta }\\ A\cap^{\;}B=C \end{array}}m_{1}\left(A\right)\cdot m_{2}\left(B\right),\forall C\in D^{\Theta }$$

where $D^{\Theta }$ is a hyperpower set from the frame $\Theta =\left\{\theta_{1},\theta_{2},\ldots ,\theta_{n}\right\}$ of *n* exhaustive elements and $A,B\in 2^{\Theta }$. The basic belief assignment is $\left(\cdot \right):2^{\Theta }\to \left[0,1\right]$, where $2^{\Theta }=\left\{\emptyset ,\theta_{1},\theta_{2},\theta_{3},\theta_{1}\cup^{\;}\theta_{2},\theta_{1}\cup^{\;}\theta_{3},\theta_{2}\cup^{\;}\theta_{3},\theta_{1}\cup^{\;}\theta_{2}\cup^{\;}\theta_{3}\right\}$ when $\Theta =\left\{\theta_{1},\theta_{2},\theta_{3}\right\}$.

In the case of the presented robot, two belief assignments were assigned to the two observers. The two observers were the force and proximity sensors that must determine which type of control was required at one time.

The experimental data for the two observers are presented in Table 2. These values divided the sensors’ measurement interval in a decision percentage, where the force sensor was more likely to decide on a dynamic control (75%) than the proximity sensor (65%). The rate was reversed for the kinematic control and was the same for the uncertain interval.

The values meant that the decision could be computed with a certain approximation by using Equation (6) if the robot was in contact with the support surface, according to the data received from the sensors, and a decision was made whether it would switch from one control type to another. The kinematic control type was used in the foot balancing phase and the dynamic control type in the support phase. The decision was made between the two contradictory objects, defined with the help of Extenics and extension theory.

Table 3 presents the cases in which the meutrosophic values *m*_1_(*θ_D_*)*, m*_1_(*θ_C_*)*, m*_2_(*θ_D_*)*, m*_2_(*θ_C_*)*, m*_1_(*θ_D_∪θ_C_*), or *m*_2_(*θ_D_∪θ_C_*) can be found in any combination for A and B to correspond to Equation (6), meaning that *A****∩****B = C*. The results were obtained using Equation (6) and represent the neutrosophic probabilistic values of truth (certainty of a valid value), falsity (assurance of a false value), uncertainty (the unknown state between two possible outcomes), and contradiction (two observers provide contradictory information with high certainty for both).

The values from Table 3 were computed in Equation (7).

(7) $$m\left(\phi \right)=0\;m\left(\theta_{D}\right)=m_{1}\left(\theta_{D}\right)\times m_{2}\left(\theta_{D}\cup^{\;}\theta_{C}\right)+m_{1}\left(\theta_{D}\cup^{\;}\theta_{C}\right)\times m_{2}\left(\theta_{D}\right)+m_{1}\left(\theta_{D}\right)\times m_{2}\left(\theta_{D}\right)=0.5575\;m\left(\theta_{C}\right)=m_{1}\left(\theta_{C}\right)\times m_{2}\left(\theta_{D}\cup^{\;}\theta_{C}\right)+m_{1}\left(\theta_{D}\cup^{\;}\theta_{C}\right)\times m_{2}\left(\theta_{C}\right)+m_{1}\left(\theta_{C}\right)\times m_{2}\left(\theta_{C}\right)=0.085\;m\left(\theta_{D}\cup^{\;}\theta_{C}\right)=m_{1}\left(\theta_{D}\cup^{\;}\theta_{C}\right)\times m_{2}\left(\theta_{D}\cup^{\;}\theta_{C}\right)=0.0025\;m\left(\theta_{D}\cap^{\;}\theta_{C}\right)=m_{1}\left(\theta_{D}\right)\times m_{2}\left(\theta_{C}\right)+m_{1}\left(\theta_{C}\right)\times m_{2}\left(\theta_{D}\right)=0.355$$

where *m*(*θ_D_*) and *m*(*θ_C_*) are the probabilistic values of certainty to choose a certain control law; *m*(*θ_D_∪θ_C_*) is the probabilistic uncertainty value of the two sensors; and *m*(*θ_D_∩θ_C_*) is the probabilistic contradiction values between the two sensors. As a test, when all five values are added, their sum must be equal to 1 (100%): *m*(*φ*) *+ m*(*θ_D_*) *+ m*(*θ_C_*) *+ m*(*θ_D_∪θ_C_*) *+ m*(*θ_D_∩θ_C_*) *=* 1.

Using the computed values, each observer’s decision (force and proximity sensor) had a certain probability that each of the two control systems required to control the robot.

**Figure 3:**
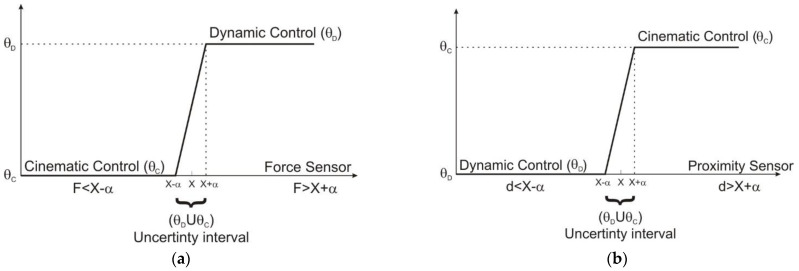
Control-deciding graphs: (**a**) force sensor graph; (**b**) proximity sensor graph.

Table 4 presents all cases presented in Figure 3a,b for the force sensor and proximity sensor where X and α were defined according to the sensor type.

For the last two cases in Table 4, the *C=* *θ_D_∪θ_C_* or *C=* *θ_D_∩θ_C_* uncertainty in decision making was due to the sensor values, leading to a contradiction. If, in the case of uncertainty, the control type running at that time could be kept, in the case of contradiction between sensor data, a decision must be made on which control should be used. Because the contradiction could appear only under specific conditions, a decision was made to use the same type of control as the robot in the case of uncertainty.

One exceptional or typical case is the robot stepping on very uneven ground. The force sensor indicates that the foot is on the floor, but the proximity sensor does not provide the same conclusion since it reads a value greater than the reference threshold. Therefore, the decision should be to switch to a dynamic controller. On the other hand, if a kinematic control is used and the foot is subject to external factors, the force sensor records high peak values in short periods, leading to the chattering effect. The algorithm switched from kinematic to dynamic control for any case of uncertainty. To prevent additional chattering effects, the algorithm switched the control method when the force sensor retained its contradictory value for a minimum Δ*t* time interval. The time threshold provided a precision control law in uneven terrain and contradictory cases between input sensors and observers.

A supplementary condition was required in addition to the selected requirements for the control type. The condition was bound to the way the robot moves. Because the dynamic control was slower to compensate for high errors and its stationary points were unnecessary, we chose the control law based on robot kinematics to save computing time.

One could argue that the switching control law is unnecessary and uses simple triggers that act as switching mechanisms. However, a simple control switch cannot decide between options when the information received is inaccurate, which is one of the main reasons the neutrosophic switching mechanism was chosen and used.

## 5. The Walking Robot Leg Control Architecture

To control a walking robot, one has to design a control law for each leg, and the control has many walking phases that depend directly on the reference signal of the foot. Therefore, the design of a general control law is needed to control foot position and the motor’s torque according to the computed reference and to use the sensor signal (force and proximity) for environmental interaction and detection.

**Figure 4:**
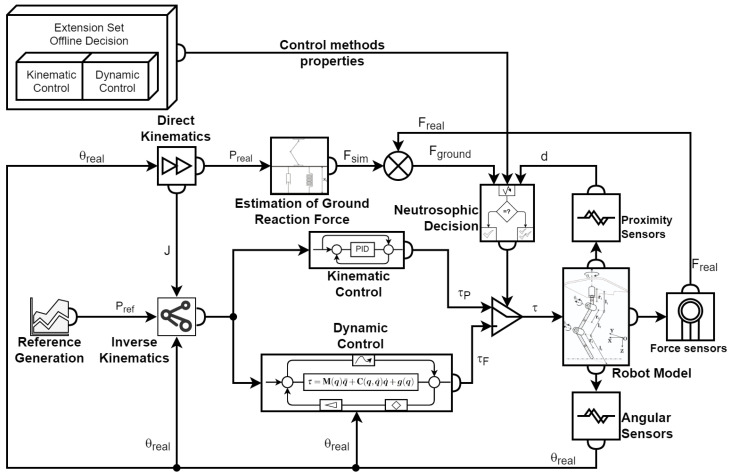
The general control diagram.

Figure 4 presents the general control diagram for one leg of the walking robot. The graph contains a reference generation block to generate the foot trajectory using detailed data chosen to test the control law.

The reference generation was made in the operational space, and the data were converted to the joint space from the operational space by using inverse kinematics. An inverse kinematics algorithm based on the Jacobian transpose was used and is presented in Equation (8). Compared to other algorithms, it provides a reference speed for the leg joint motors and not the angular position reference.

(8) $$\begin{array}{cc} 1. & \Delta e=e_{goal}-e_{real}\\ 2. & JJTde=J\times J^{T}\times \Delta e\\ 3. & \alpha =\frac{\Delta e^{T}\times JJTde}{JJTde^{T}\times JJTde}\\ 4. & \Delta \theta ={\left(\alpha \times J^{T}\times \Delta e\right)}^{T} \end{array}.$$

The speed reference value cannot be used to control the robot joints by the dynamic controller because the dynamic controller needs the angular reference for all the degrees of freedom it controls, and this is the reason why the angular values for each joint were computed using the foot position as the origin. The equations are:

(9) $$\begin{array}{c} q_{1}=\arctan\left(\frac{Mx}{My}\right)\\ q_{2}=2\times \arctan\left(\frac{\sin q_{2}}{\sin^{2}q_{2}+\cos^{2}q_{2}}+\cos q_{2}\right)\\ q_{3}=\arctan\left(-\frac{\sin q_{3}}{\cos q_{3}}\right) \end{array}$$

where the sine and cosine values are given by

(10) $$\cos q_{2}=\frac{\left(Mz-l_{1}\right)\times \left(l_{2}+l_{3}\cos q_{3}\right)+Mx\times l_{3}\sin q_{3}}{{\left(Mz-l_{1}\right)}^{2}+Mx^{2}+My^{2}},\;\sin q_{2}=\sqrt{1-\cos^{2}q_{2}},\;\cos q_{3}=\frac{{\left(Mz-l_{1}\right)}^{2}+Mx^{2}+My^{2}-l_{2}^{2}-l_{3}^{2}}{2l_{2}l_{3}},\;\sin q_{3}=\sqrt{1-\cos^{2}q_{3}}.$$

For Equation (9) to be valid and to condition the leg posture, additional conditions were added:

(11) $$\begin{array}{cc} 1: & if\;\;\mathrm{My}=0\;then\;\;\;q1=0\\ 2: & \;q_{1}\in \left(-\frac{\pi }{2},\frac{\pi }{2}\right)\\ 3: & q_{3}\leq 0 \end{array}.$$

**Figure 5:**
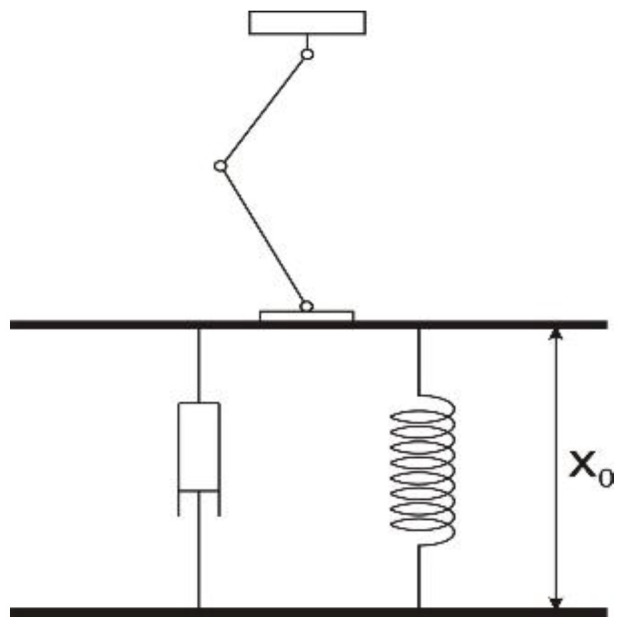
Interaction between the robot and ground surface.

The two sensors’ data (proximity and force) were used as input signals for the neutrosophic block to decide. Because generated information was used, the two sensors were simulated. Therefore, the proximity sensor had a function based on the calculated distance from the foot to the support surface considered a plane, but to which a sinusoidal signal was added to generate the measurement error of the sensor. Regarding the force sensor, the foot–ground interaction was simulated using the system from Figure 5. The simulation was achieved with the help of a damper and a spring.

The equation used for contract modeling and determining the reaction force of ground interaction was the classical one:

(12) $$F_{tot}=-\left(k\cdot x+c\cdot \dot{x}\right)$$

where *k* and ***c*** are the constants of the spring and damper, respectively.

Having the two parameters, reaction force and proximity distance, as inputs for the decision method, the two control methods are defined in the following sections.

### 5.1. The Kinematic Control Method

**Figure 6:**
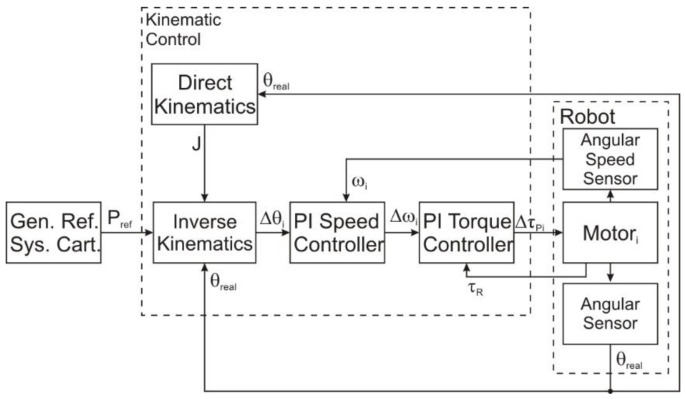
The kinematic control diagram.

This method used the data provided by the computing algorithm of inverse kinematics (Figure 6) and fed the output to the PI (proportional-integration) regulator that drove the robot joint motors.

As previously described in the Extenics method, the control method has fewer calculations. Still, the positioning error is not the best because of the inverse kinematics method. It does not consider the inertial force that the robot experiences during the actual motion.

The main component of the controller is the Jacobian matrix:

(13) $$J=\left[\begin{array}{ccc} -s_{1}(l_{2}s_{2}+l_{3}s_{23}) & c_{1}\left(l_{2}c_{2}+l_{3}c_{23}\right) & l_{3}c_{1}c_{23}\\ c_{1}\left(l_{2}s_{2}+l_{3}s_{23}\right) & s_{1}\left(l_{2}c_{2}+l_{3}c_{23}\right) & l_{3}s_{1}c_{23}\\ 0 & -l_{2}s_{2}-l_{3}s_{23} & -l_{3}s_{23} \end{array}\right]$$

where *s_i_* = sin(*θ_i_*), *c_i_* = cos(*θ_i_*), *s_ij_* = sin(*θ_i_* + *θ_j_*), and *c_ij_* = cos(*θ_i_* + *θ_j_*).

The matrix was computed from the direct kinematics equations and was used to find the foot position in the operational space:

(14) $$M\left(x,y,z\right)=\left[\begin{array}{c} c_{1}\left(l_{2}s_{2}+l_{3}s_{23}\right)\\ s_{1}\left(l_{2}s_{2}+l_{3}s_{23}\right)\\ l_{1}+l_{2}c_{2}+l_{3}c_{23} \end{array}\right].$$

The entire kinematic control loop was based on the Jacobian matrix. First, the matrix containing the actual angular joint position was calculated. After it followed its transpose matrix and the operational space reference position, the positioning error Δ*θ* was obtained.

The positioning error was sent to two PI (proportional-integrative) feedback control loops for controlling the angular speed and motor torque. Thereby, the torque control for each joint was obtained, and the switch from one controller to another (from the kinematic control to the dynamic one, and vice versa) was more accessible since they both used torque to control the robot joints.

The transpose Jacobian method is not new and is based on using the transpose matrix of the Jacobian instead of the inverse matrix. Therefore, Δ*θ* was computed using Equation (15):

(15) $$\Delta \dot{q}=\alpha J^{T}e$$

for specific values of constant α.

The transpose-Jacobian-matrix-based algorithm presented in Equation (8) eliminated stability problems. The algorithm was also chosen because it had a higher computation speed than the control values of other algorithms, even if the computed values were not as precise as the inverse-Jacobian-matrix-based method [44].

Because the method of solving the inverse kinematics problem uses the Jacobian matrix, the final results are always formed by angular speeds that the robot joints must follow. Therefore, the control is suitable for PI and PID regulators and for controlling angular velocities. The downside is that the method cannot be used to compute a dynamic control reference since it needs a precise joint angular value. In contrast, if the values given by the Jacobian-based inverse kinematic problem are integrated, the result is not as accurate as is required.

### 5.2. The Dynamic Control Method

The dynamic control method used the same reference data as the kinematic one. Nevertheless, it computed the torque reference of the motors considering kinematic parameters, the inertial ones provided by the inertia matrix, and the Coriolis and gravity force effects supplied by the Coriolis and gravity matrices.

**Figure 7:**
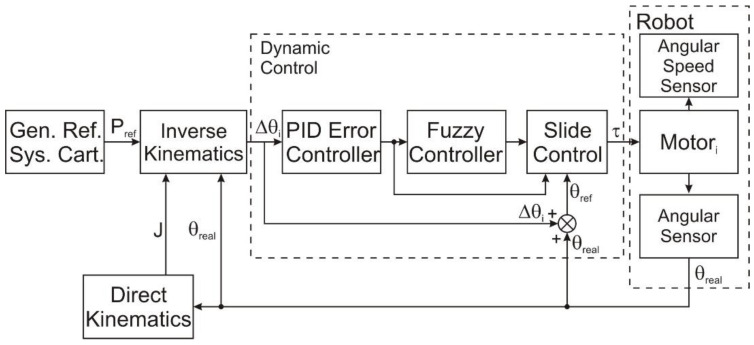
The dynamic control scheme.

**Figure 8:**
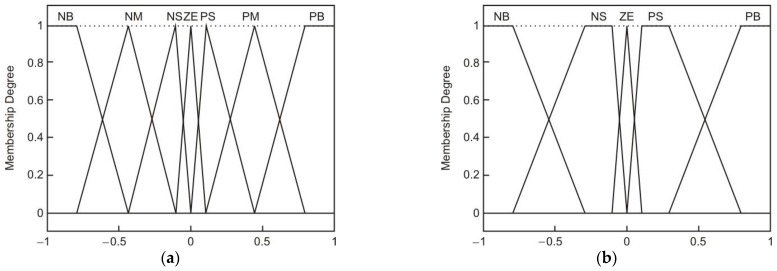
Membership functions: (**a**) member function for input s; (**b**) member function for input $\dot{s}$.

Figure 7 presents the dynamic control diagram. The most critical control blocks are shown, including those that compute the inertial parameters and values used by the slide control block. The three control blocks that formed the dynamic controller from Figure 7 were the PID error controller, the fuzzy controller, and the slide control. The first block passed the positioning error through a PID controller so that the control method could consider the error variations. Using the PID error controller data, the fuzzy amplification was obtained through the membership functions presented in Figure 8a,b. The command torque for each motor joint could be calculated after computing the fuzzy gain, using the inertial data and the reference values [45].

All the control values were computed from the presented robot structure, characteristics, structural weights, and measurements.

For Figure 8a,b the abbreviations are N = Negative, P = Positive, ZE = zero, S = Small, M = Medium, B = Big, and V = Very. For the membership functions, Table 5 presents the values for the membership parameters so the gain value *K_fuzzy_* could be chosen. The membership functions that provided the gain were selected according to the values of the two parameters *s* and $\dot{s}$, where *s* represents the error through the PID error controller and $\dot{s}$ is its derivate. A constant gain was not desired for each case, leading to a standard-step fuzzy controller, but a function-based one was selected.

**Figure 9:**
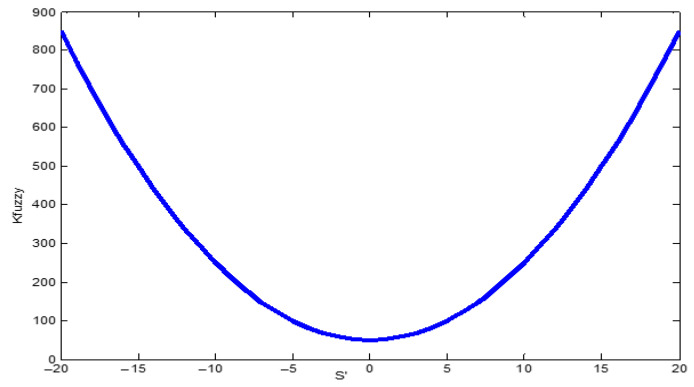
Parameter *K_fuzzy_* for *s = 0* and $\dot{s}$ between −20 and 20.

Using Table 5 data, the parabola in Figure 9 was considered for computing the *K_fuzzy_* gain, according to the two inputs *s* and $\dot{s}$. The parabola equation was computed from Equation (16):

(16) $$y\left(x\right)=2x^{2}+50,$$

and we modified it to introduce the fuzzy parameters:

(17) $$K_{fuzzy}\left(\dot{s}\right)=2{\left(\dot{s}-10\cdot s\right)}^{2}+50.$$

Equation (15) now provides the *K_fuzzy_* parameter in the dynamic control.

The sliding control was made with the help of the slide control block (Figure 7). The control type was inspired by Shafiei [12] and modified to match the robot kinematic structure used, a design with three degrees of freedom instead of the two used by Shafiei [12]. Following that, the dynamic equations that allowed the dynamic controller’s development are presented.

The basic dynamic control equation was:

(18) $$H\left(q\right)\ddot{q}+C\left(q,\dot{q}\right)\dot{q}+G\left(q\right)+\tau_{d}=\tau .$$

From Equation (18), the signal for motor torque control was calculated. All the parameters from Equation (18) are required to be known. The unknown values are the torque *τ*, the matrices *H* (inertial parameters), *C* (Coriolis and centrifugal forces), and *G* (gravity effect), which are given by the following equations, in which the angles *θ* from the joint space are equal to the ones in the operational space:

(19) $$H=M=\left[\begin{array}{ccc} M_{11} & M_{12} & M_{13}\\ M_{21} & M_{22} & M_{23}\\ M_{31} & M_{32} & M_{33} \end{array}\right]$$

where *M* is the inertial parameters matrix.

The inertial matrix parameters can be computed using the following:

(20) $$T\left(\theta ,\dot{\theta }\right)=\frac{1}{2}{\dot{q}}^{T}\cdot M\cdot \dot{q}=\frac{1}{2}\sum_{i,j}M_{ij}\left(q\right){\dot{q}}_{i}{\dot{q}}_{j}\geq 0,\;T(\theta ,\dot{\theta })=\frac{1}{2}m_{1}\left({\dot{\overset{-}{x}}}_{1}^{2}+{\dot{\overset{-}{y}}}_{1}^{2}+{\dot{\overset{-}{z}}}_{1}^{2}\right)+\frac{1}{2}m_{2}\left({\dot{\overset{-}{x}}}_{2}^{2}+{\dot{\overset{-}{y}}}_{2}^{2}+{\dot{\overset{-}{z}}}_{2}^{2}\right)+\frac{1}{2}m_{3}\left({\dot{\overset{-}{x}}}_{3}^{2}+{\dot{\overset{-}{y}}}_{3}^{2}+{\dot{\overset{-}{z}}}_{3}^{2}\right)+\frac{1}{2}I_{z_{1}}\dot{\theta_{1}^{2}}+\frac{1}{2}I_{z_{2}}{\left(\dot{\theta_{1}}+\dot{\theta_{2}}\right)}^{2}+\;\frac{1}{2}I_{z_{3}}{\left(\dot{\theta_{1}}+\dot{\theta_{2}}+\dot{\theta_{3}}\right)}^{2}\;{\overset{-}{x}}_{1}=0,\;{\dot{\overset{-}{x}}}_{1}=0,\;{\overset{-}{y}}_{1}=0,{\dot{\overset{-}{y}}}_{1}=0,\;{\overset{-}{z}}_{1}=r_{1},\;{\dot{\overset{-}{z}}}_{1}=0,\;\begin{array}{ll} {\overset{-}{x}}_{2}=r_{2}\sin q_{2}\cdot \cos q_{1}, & {\dot{\overset{-}{x}}}_{2}=-r_{2}\sin q_{1}-\sin q_{2}\cdot {\dot{q}}_{1}+r_{2}\cos q_{1}\cdot \cos q_{2}\cdot {\dot{q}}_{2},\\ {\overset{-}{y}}_{2}=r_{2}\sin q_{2}\cdot \sin q_{1}, & {\dot{y}}_{2}=r_{2}\cos q_{1}\cdot \sin q_{2}\cdot {\dot{q}}_{1}+r_{2}\sin q_{1}\cdot \cos q_{2}\cdot {\dot{q}}_{2},\\ {\overset{-}{z}}_{2}=l_{1}+r_{2}\cos q_{2}, & {\dot{z}}_{2}=-r_{2}\sin q_{2}\cdot {\dot{q}}_{2}, \end{array}\;{\overset{-}{x}}_{3}=\cos q_{1}\left(l_{2}\sin q_{2}+r_{3}\sin\left(q_{2}+q_{3}\right)\right),\;{\overset{-}{y}}_{3}=\sin q_{1}\left(l_{2}\sin q_{2}+r_{3}\sin\left(q_{2}+q_{3}\right)\right),\;{\overset{-}{z}}_{3}=l_{1}+l_{2}\cos q_{2}+r_{3}\cos\left(q_{2}+q_{3}\right),\;{\dot{\overset{-}{x}}}_{3}=-\sin q_{1}\left(l_{2}\sin q_{2}+r_{3}\sin\left(q_{2}+q_{3}\right)\right){\dot{q}}_{1}+\cos q_{1}\left(l_{2}\cos q_{2}+r_{3}\cos\left(q_{2}+q_{3}\right)\right){\dot{q}}_{2}+r_{3}\cos q_{1}\cos\left(q_{2}+q_{3}\right){\dot{q}}_{3},\;{\dot{\overset{-}{y}}}_{3}=\cos q_{1}\left(l_{2}\sin q_{2}+r_{3}\sin\left(q_{2}+q_{3}\right)\right){\dot{q}}_{1}+\sin q_{1}\left(l_{2}\cos q_{2}+r_{3}\cos\left(q_{2}+q_{3}\right)\right){\dot{q}}_{2}+r_{3}\sin q_{1}\cos\left(q_{2}+q_{3}\right){\dot{q}}_{3}\;{\dot{\overset{-}{z}}}_{3}=-\left(l_{2}\sin q_{2}+r_{3}\sin\left(q_{2}+q_{3}\right)\right){\dot{q}}_{2}-r_{3}\sin\left(q_{2}+q_{3}\right){\dot{q}}_{3},\;I_{xi}=\frac{m_{i}}{12}\left(w_{i}^{2}+h_{i}^{2}\right),\;I_{yi}=\frac{m_{i}}{12}\left(l_{i}^{2}+h_{i}^{2}\right),\;I_{zi}=\frac{m_{i}}{12}\left(l_{i}^{2}+w_{i}^{2}\right)$$

where ${\overset{-}{x}}_{i},{\overset{-}{y}}_{i},{\overset{-}{z}}_{i},{\dot{\overset{-}{x}}}_{i},{\dot{\overset{-}{y}}}_{i},{\dot{\overset{-}{z}}}_{i}$ are the coordinates of the center of mass for each element of a leg, and, respectively, their first derivate; and *I_xi_*, *I_yi_*, and *I_zi_* represent the inertia tensors of each leg element.

By using inertia matrix requirement parameters, the inertia matrix elements were computed by the following equations:

(21) $$M_{11}=r_{2}^{2}m_{2}+r_{3}^{2}m_{3}+l_{2}^{2}m_{3}+\frac{1}{12}l_{1}^{2}m_{1}+\frac{1}{12}w^{2}m_{1}+\frac{1}{12}l_{2}^{2}m_{2}+\frac{1}{12}w^{2}m_{2}+\frac{1}{12}l_{3}^{2}m_{3}+\frac{1}{12}w^{2}m_{3}+\;2l_{2}r_{3}m_{3}\sin\left(q_{2}+q_{3}\right)-\cos^{2}\left(q_{2}\right)\left(l_{2}^{2}m_{3}+r_{2}^{2}m_{2}\right)-r_{3}^{2}m_{3}\cos^{2}\left(q_{2}+q_{3}\right),\;M_{12}=\frac{1}{6}l_{2}^{2}m_{2}+\frac{1}{6}w^{2}m_{2},\;M_{21}=\frac{1}{6}w^{2}m_{3}+\frac{1}{6}l_{3}^{2}m_{3},\;M_{22}=r_{2}^{2}m_{2}+l_{2}^{2}m_{3}+r_{3}^{2}m_{3}+\frac{1}{12}w^{2}m_{2}+\frac{1}{12}l_{2}^{2}m_{2}+\frac{1}{12}l_{3}^{2}m_{3}+\frac{1}{12}w^{2}m_{3}+2l_{2}r_{3}m_{3}\left[\sin q_{2}\sin\left(q_{2}+q_{3}\right)+\;\cos q_{2}\cos\left(q_{2}+q_{3}\right)\right],\;M_{23}=2l_{2}r_{3}m_{3}\left[\sin q_{2}\cdot \sin\left(q_{2}+q_{3}\right)+\cos q_{2}\cdot \cos\left(q_{2}+q_{3}\right)\right],\;M_{31}=\frac{1}{6}l_{3}^{2}m_{3},\;M_{32}=\frac{1}{6}w^{2}m_{3}+\frac{1}{6}l_{3}^{2}m_{3}+2r_{3}^{2}m_{3},\;M_{33}=r_{3}^{2}m_{3}+\frac{1}{12}l_{3}^{2}m_{3}+\frac{1}{12}w^{2}m_{3}.$$

The Coriolis matrix was computed using the following equation:

(22) $$C_{ij}\left(q,\dot{q}\right)=\sum_{k=1}^{3}\Gamma_{ijk}{\dot{q}}_{k}=\frac{1}{2}\sum_{k=1}^{3}\left(\frac{\partial M_{ij}}{\partial q_{k}}+\frac{\partial M_{ik}}{\partial q_{j}}-\frac{\partial M_{kj}}{\partial q_{i}}\right){\dot{q}}_{k}$$

for which the *Γ_ijk_* parameters are:

(23) $$\Gamma_{111}=\Gamma_{122}=\Gamma_{123}=\Gamma_{132}=\Gamma_{133}=\Gamma_{212}=\Gamma_{213}=\Gamma_{221}=\Gamma_{222}=\Gamma_{231}=\Gamma_{312}=\Gamma_{313}=\Gamma_{321}=\Gamma_{323}=\Gamma_{331}=\Gamma_{333}=0,\;\Gamma_{112}=\left(\begin{array}{c} 2l_{2}m_{3}r_{3}\left(\sin q_{2}\cos\left(q_{2}+q_{3}\right)+\cos q_{2}\sin\left(q_{2}+q_{3}\right)\right)+2l_{2}^{2}m_{3}\cos q_{2}\sin q_{2}+\\ +2m_{2}r_{2}^{2}\cos q_{2}\sin q_{2}+2m_{3}r_{3}^{2}\cos\left(q_{2}+q_{3}\right)\sin\left(q_{2}+q_{3}\right) \end{array}\right){\dot{q}}_{2},\;\Gamma_{113}=\left(2l_{2}m_{3}\sin q_{2}\cos\left(q_{2}+q_{3}\right)+2m_{3}r_{3}^{2}\cos\left(q_{2}+q_{3}\right)\sin\left(q_{2}+q_{3}\right)\right){\dot{q}}_{3}\;\Gamma_{121}=\left(\begin{array}{c} 2l_{2}m_{3}r_{3}\left(\sin q_{2}\cos\left(q_{2}+q_{3}\right)+\cos q_{2}\sin\left(q_{2}+q_{3}\right)\right)+2l_{2}^{2}m_{3}\cos q_{2}\sin q_{2}+\\ +2m_{2}r_{2}^{2}\cos q_{2}\sin q_{2}+2m_{3}r_{3}^{2}\cos\left(q_{2}+q_{3}\right)\sin\left(q_{2}+q_{3}\right) \end{array}\right){\dot{q}}_{1},\;\Gamma_{131}=\left(2l_{2}m_{3}\sin q_{2}\cos\left(q_{2}+q_{3}\right)+2m_{3}r_{3}^{2}\cos\left(q_{2}+q_{3}\right)\sin\left(q_{2}+q_{3}\right)\right){\dot{q}}_{1}\;\Gamma_{211}=-\left(\begin{array}{c} 2l_{2}m_{3}r_{3}\left(\sin q_{2}\cos\left(q_{2}+q_{3}\right)+\cos q_{2}\sin\left(q_{2}+q_{3}\right)\right)+2l_{2}^{2}m_{3}\cos q_{2}\sin q_{2}+\\ +2m_{2}r_{2}^{2}\cos q_{2}\sin q_{2}+2m_{3}r_{3}^{2}\cos\left(q_{2}+q_{3}\right)\sin\left(q_{2}+q_{3}\right) \end{array}\right){\dot{q}}_{1},\;\Gamma_{223}=2l_{2}m_{3}r_{3}\left(\sin q_{2}\cos\left(q_{2}+q_{3}\right)-\cos q_{2}\sin\left(q_{2}+q_{3}\right)\right){\dot{q}}_{3},\;\Gamma_{232}=2l_{2}m_{3}r_{3}\left(\sin q_{2}\cos\left(q_{2}+q_{3}\right)-\cos q_{2}\sin\left(q_{2}+q_{3}\right)\right){\dot{q}}_{2},\;\Gamma_{233}=4l_{2}m_{3}r_{3}\left(\sin q_{2}\cos\left(q_{2}+q_{3}\right)-\cos q_{2}\sin\left(q_{2}+q_{3}\right)\right){\dot{q}}_{3},\;\Gamma_{311}=-\left(2l_{2}m_{3}\sin q_{2}\cos\left(q_{2}+q_{3}\right)+2m_{3}r_{3}^{2}\cos\left(q_{2}+q_{3}\right)\sin\left(q_{2}+q_{3}\right)\right){\dot{q}}_{1},\;\Gamma_{322}=-2l_{2}m_{3}r_{3}\left(\sin q_{2}\cos\left(q_{2}+q_{3}\right)-\cos q_{2}\sin\left(q_{2}+q_{3}\right)\right){\dot{q}}_{2},\;\Gamma_{332}=-2l_{2}m_{3}r_{3}\left(\sin q_{2}\cos\left(q_{2}+q_{3}\right)-\cos q_{2}\sin\left(q_{2}+q_{3}\right)\right){\dot{q}}_{2}.$$

The last part of the dynamic equation is given by Equation (24), which computed the gravity effect matrix on the robot leg:

(24) $$G\left(q\right)=N\left(q,\dot{q}\right)=\frac{\partial U}{\partial q}=\left[\begin{array}{c} \frac{\partial U}{\partial q_{1}}\\ \frac{\partial U}{\partial q_{2}}\\ \frac{\partial U}{\partial q_{3}} \end{array}\right]$$

where

(25) $$\begin{array}{c} U(q)=m_{1}x_{1}g+m_{1}y_{1}g+m_{1}z_{1}g+m_{2}g\left(x_{2}+y_{2}+z_{2}\right)+m_{3}g\left(x_{3}+y_{3}+z_{3}\right)\\ =m_{1}r_{1}g+m_{2}g\left(l_{1}+r_{2}\cos q_{2}+r_{2}\sin q_{2}\left(\sin q_{1}+\cos q_{1}\right)\right)+\\ +m_{3}g\left(l_{1}+l_{2}\cos q_{2}+r_{3}\cos\left(q_{2}+q_{3}\right)+\left(\sin q_{1}+\cos q_{1}\right)\left(l_{2}\sin q_{2}+r_{3}\sin\left(q_{2}+q_{3}\right)\right)\right) \end{array}$$

and its derivative components are:

(26) $$\frac{\partial U}{\partial q_{1}}=m_{2}r_{2}g\sin q_{2}\left(-\sin q_{1}+\cos q_{1}\right)+m_{3}g\left(-\sin q_{1}+\cos q_{1}\right)\left(l_{2}\sin q_{2}+r_{3}\sin\left(q_{2}+q_{3}\right)\right)\;\frac{\partial U}{\partial q_{2}}=m_{2}g\left(-r_{2}\sin q_{2}+r_{2}\cos q_{2}\left(\cos q_{1}+\sin q_{1}\right)\right)\;+m_{3}g\left[-l_{2}\sin q_{2}-r_{3}\sin\left(q_{2}+q_{3}\right)+\left(\sin q_{1}+\cos q_{1}\right)\left(l_{2}\cos q_{2}+r_{3}\cos\left(q_{2}+q_{3}\right)\right)\right]\;\frac{\partial U}{\partial q_{3}}=m_{3}g\left[-r_{3}\sin\left(q_{2}+q_{3}\right)+r_{3}\cos\left(q_{2}+q_{3}\right)\left(\sin q_{1}+\cos q_{1}\right)\right].$$

Using the dynamic control equations, the PID sliding control can be developed, which computes the torque *τ* used in the joint motor torque control so that the position vector *q* can track the desired trajectory *q_d_*. The tracking error vector is defined as:

(27) $$e=q_{d}-q.$$

Sliding motion control requires a sliding surface, given by Equation (28), and contains both the derivative term and the integral one:

(28) $$s=\dot{e}+\lambda_{1}e+\lambda_{2}\int_{0}^{t}edt$$

where *λ_i_* is a positive diagonal matrix; it turns out that, for *s* = 0, a stable sliding surface is obtained (as shown by Shafiei in [12]). The dynamic robot equations can be written by using the sliding surface equation:

(29) $$H\dot{s}=-Cs+f+\tau_{d}-\tau$$

where

(30) $$f=H\left({\ddot{q}}_{d}+\lambda_{1}\dot{e}+\lambda_{2}e\right)+C\left({\dot{q}}_{d}+\lambda_{1}e+\lambda_{2}\int_{0}^{t}edt\right)+G.$$

The control module input becomes:

(31) $$\tau =\hat{f}+K_{v}s+K\mathrm{sgn}\left(s\right)$$

where

(32) $$\hat{f}=\hat{H}\left({\ddot{q}}_{d}+\lambda_{1}\dot{e}+\lambda_{2}e\right)+\hat{C}\left({\dot{q}}_{d}+\lambda_{1}e+\lambda_{2}\int_{0}^{t}edt\right)+\hat{G}.$$

Equation (32) represents a force estimation *f*, and $K_{v}s=K_{v}\dot{e}+K_{v}\lambda_{1}e+K_{v}\lambda_{2}\int_{0}^{t}edt$ is the outer PID loop; *K_v_* and *K* are positive diagonal matrices built so that the stability conditions are fulfilled and guaranteed. The *sgn*(*s*) function is the sign function. The function can also be written as:

(33) $$\left|\tilde{f}\right|=\left|\tilde{H}\left({\ddot{q}}_{d}+\lambda_{1}\dot{e}+\lambda_{2}e\right)+\tilde{C}\left({\dot{q}}_{d}+\lambda_{1}e+\lambda_{2}\int_{0}^{t}e\;dt\right)+\tilde{G}\right|\leq F$$

where $\tilde{f}=f-\hat{f}$, $\tilde{H}=H-\hat{H}$, and $\tilde{G}=G-\hat{G}$. The vector *F* is:

(34) $$F=\left|\tilde{H}\left({\ddot{q}}_{d}+\lambda_{1}\dot{e}+\lambda_{2}e\right)\right|+\left|\tilde{C}\left({\dot{q}}_{d}+\lambda_{1}e+\lambda_{2}\int_{0}^{t}e\;dt\right)\right|+\left|\tilde{G}\right|.$$

To control the system states $\left(e,\dot{e}\right)$ and to reach the sliding surface *s =* 0 in a limited time by staying on the surface, the control law should be formulated so that Condition (35) is fulfilled:

(35) 12ddt[sTHs]<η(sTs)12, η>0.

Using the sign function in the control law, high oscillations in the control torque are found as the undesired phenomenon called chattering. To overcome this drawback, a saturation function was used (36) for the discontinuous part of the control law:

(36) $$sat\left(\frac{s}{\varphi }\right)=\left\{\begin{array}{cc} 1 & s\geq \varphi \\ \frac{s}{\varphi } & -\varphi <s<\varphi \\ -1 & s\leq -\varphi \end{array}\right\}.$$

As a result, a layer *ϕ* around the sliding surface was obtained so that, when the robot foot trajectory was inside the layer, it remained there. The values of *λ*_1_, *λ*_2_, *K,* and *K_v_* were adjusted to better position the mobile walking robot foot.

## 6. Hybrid Control Simulation

**Figure 10:**
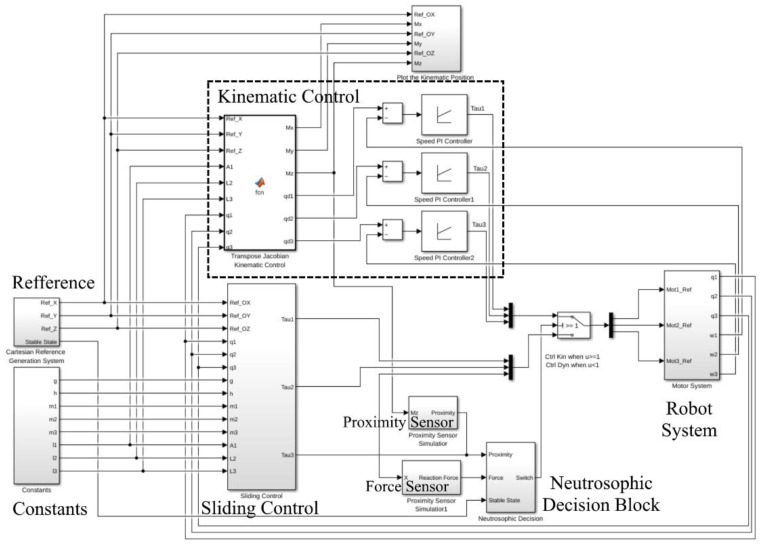
MATLAB Simulink diagram for the hybrid control.

**Figure 11:**
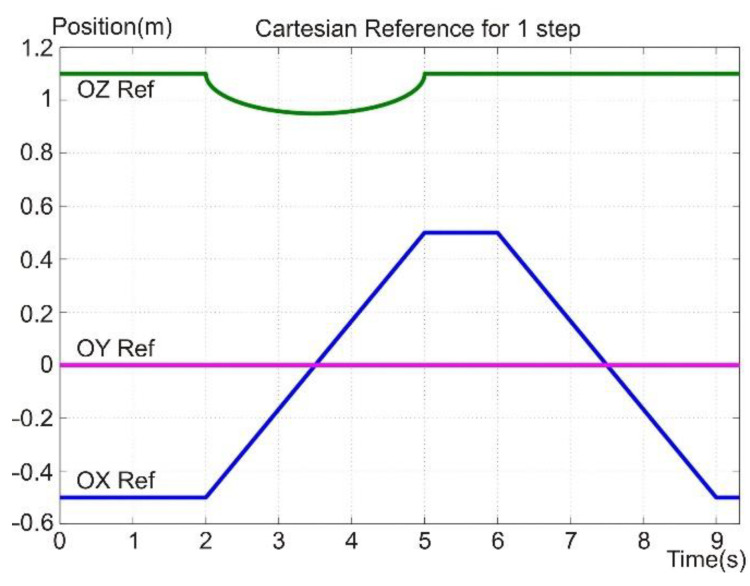
Foot reference signals.

**Figure 12:**
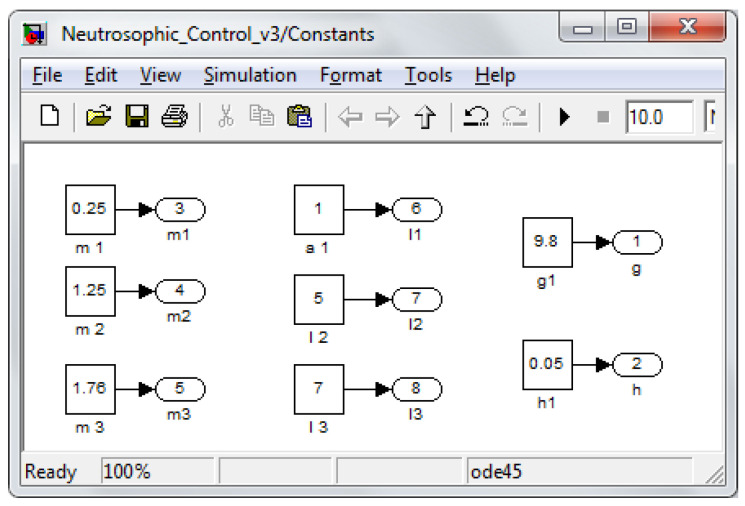
Constant values.

The simulation was built with the help of MATLAB Simulink software to test the proposed methods and control laws. Figure 10 presents the diagram of the main components of the hybrid controller. The reference generation block for the OXYZ axis in the Cartesian space is shown in Figure 11, and the constant generation block defining the walking robot is presented in Figure 12. All the values were sent to a reference system on the robot structure, illustrated in Figure 1b. The foot’s vertical position was at a distance of 1.1 m from the origin set on the robot platform, not the foot.

The three lines in Figure 11 represent the reference system as follows: the top signal (green line) is the reference for the robot foot on the OZ axis, the trapezoidal signal (blue line) is the reference for the robot foot on the OX axis, and for the OY axis, a zero-value signal was used (purple line). These three datasets represent the Cartesian position of the robot foot for a complete cycle of a leg’s walking step. The reference on the OY axis is the heading direction of the robot and has a trapezoidal shape because the foot is moving relative to the robot platform.

**Figure 13:**
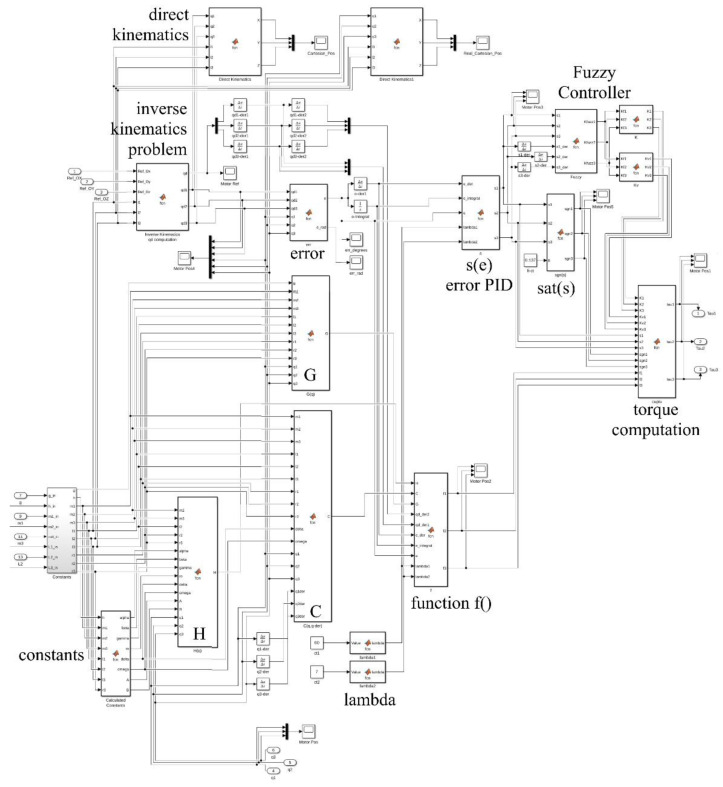
The sliding control diagram made in MATLAB Simulink.

Figure 13 shows the diagram corresponding to the sliding control method made in MATLAB Simulink in which all the described elements are found. With their help, the command signal for the three joint motors was calculated.

Algorithm 1 controlled the kinematic control block. It computed the angular speeds using the Jacobian matrix and the formula from Equation (8), which provided the angular reference speed.
**Algorithm 1** The kinematic control blockfunction [Mx, My, Mz, qd]=fcn(Ref_X, Ref_Y, Ref_Z, A1, L2, L3, q1, q2, q3) fi1=q1; fi2=q2; fi3=q3; J11=−sind(fi1)×(L2×sind(fi2)+L3×sind(fi2+fi3)); J12=L2×cosd(fi1)×cosd(fi2)+L3×cosd(fi1)×cosd(fi2+fi3); J13=L3×cosd(fi1)×cosd(fi2+fi3); J21=cosd(fi1)×(L2×sind(fi2)+L3×sind(fi2+fi3)); J22=L2×sind(fi1)×cosd(fi2)+L3×sind(fi1)×cosd(fi2+fi3); J23=L3×sind(fi1)×cosd(fi2+fi3); J31=0; J32=−L2×sind(fi2) −L3×sind(fi2+fi3); J33=−L3×sind(fi2+fi3); Jb=[J11 J12 J13; J21 J22 J23; J31 J32 J33]; Mx=cosd(fi1)×(L2×sind(fi2)+L3×sind(fi2+fi3)); My=sind(fi1)×(L2×sind(fi2)+L3×sind(fi2+fi3)); Mz=L2×cosd(fi2)+L3×cosd(fi2+fi3)+A1; M_err=[Ref_X−Mx; Ref_Y−My; Ref_Z−Mz]; $\mathrm{JJTde}=\mathrm{Jb}\times {\mathrm{Jb}}^{\prime }\mathrm{xM}\_\mathrm{err};$ $\mathrm{qd}=\mathrm{alpha}\;\times {\mathrm{Jb}}^{\prime }\times \;M\_\mathrm{err};$

**Figure 14:**
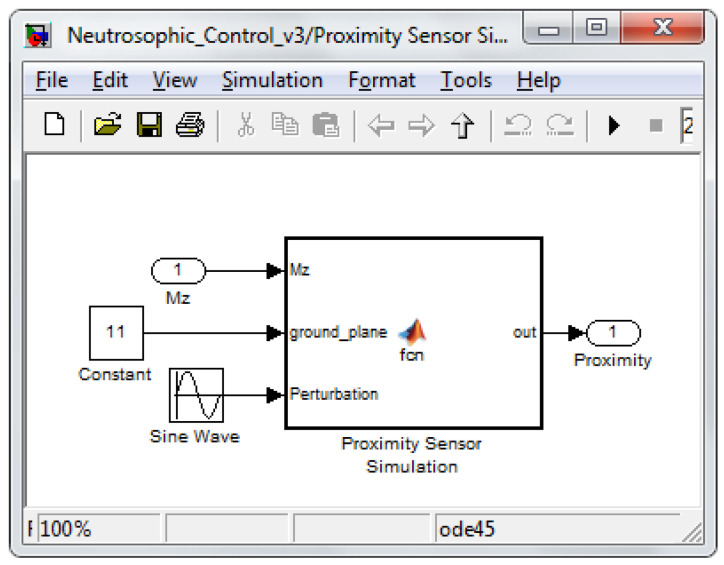
Proximity sensor simulation.

**Figure 15:**
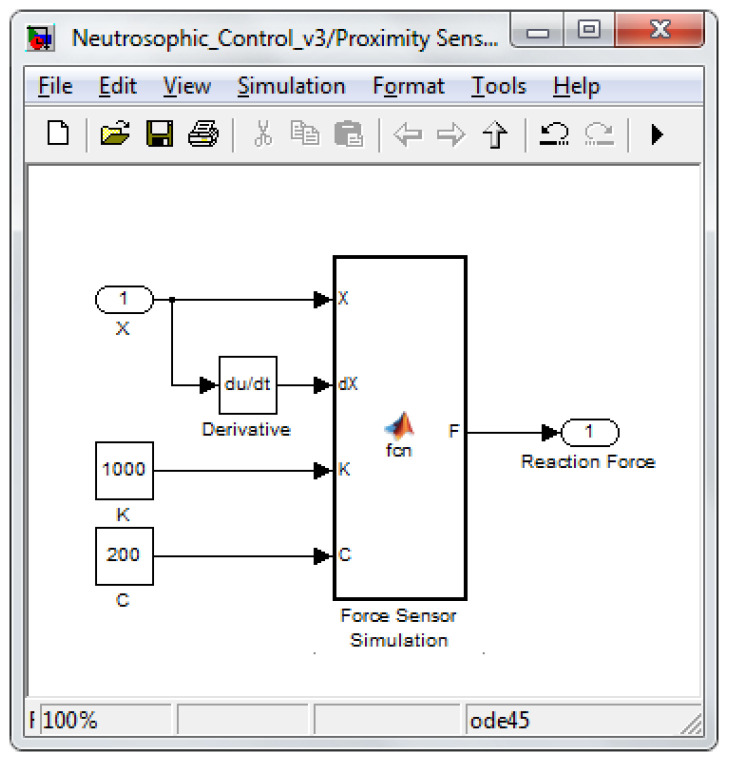
Force sensor simulation.

Figure 14 and Figure 15 present the simulation diagrams for the two sensors used in determining which control law should be used at a particular moment in time according to the switching algorithm based on neutrosophic logic.

**Figure 16:**
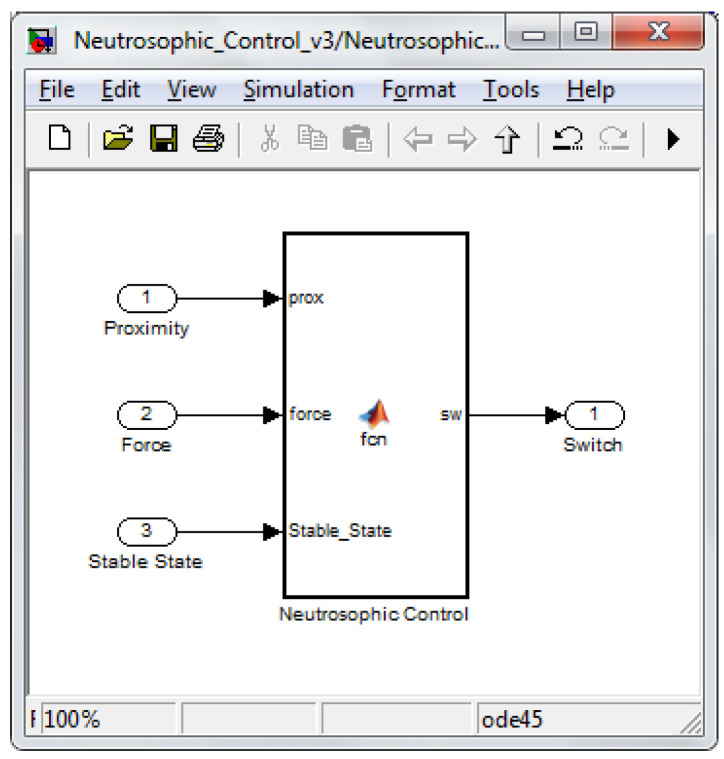
Neutrosophic switching diagram.

Using what was presented in Section 4 regarding the neutrosophic decision, the neutrosophic control switching block was implemented. It is illustrated in Figure 16 with its inputs and outputs. The two inputs already described are shown, bringing proximity and force information into the switching mechanism. In addition to these two, there was a third input called stable-state, and it provided the block with additional information. When the robot was homing or reached specific points, it was controlled only by the kinematic control law. The solution was chosen to save computing power and provide a higher speed for arriving at the initial position (homing phase).

The actual neutrosophic switching block followed the detailed conditions already described.

## 7. Experimental Results 

Following the simulation results, we observed several things. One of them was that, to successfully simulate the control law, which was bounded by the interaction between the support surface and the robot, different conditions were needed by the decision and control methods. This case was observed during the support phase, for which the robot leg must hold the entire robot weight and carry out the forward robot motion. The force and proximity sensors must have values that assumed the support surface contact in actual case conditions. In contrast, in simulation conditions, if the positioning control error placed the robot foot slightly above the support surface, then the sensors could affect the control laws and the entire system. Consequently, the switching mechanism was built with the condition that switched the control law when there was permanent contact with the support surface. An example is the homing motion of the foot, for which the robot was controlled only through the kinematic control method.

**Figure 17:**
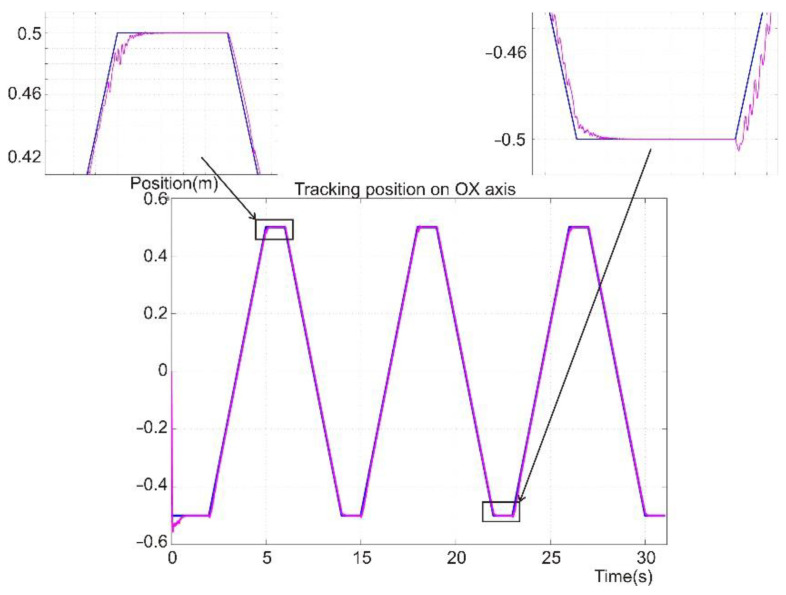
Foot reference and position tracking on the OX axis.

**Figure 18:**
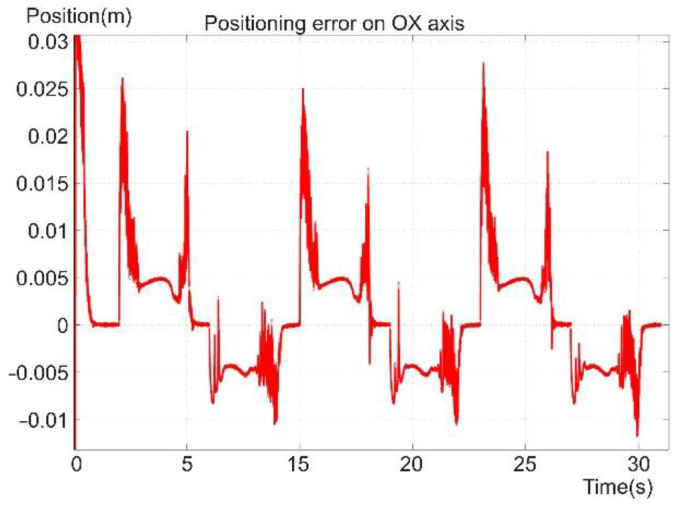
Foot positioning error on the OX axis.

Figure 17 presents the reference and position tracking for the robot foot in the operational space in Cartesian coordinates on the OX axis. The positioning error on the OX axis is shown in Figure 18. The movement represents the forward direction of motion for the robot and its legs. Thus, three steps are presented, for which the trapezoidal shape of the signal represents the forward and retreat motion relative to the robot platform. The movement was computed according to the reference system relative to the robot platform. Because the reference was considered in the robot’s operational space, the first coordinate system was selected at the point where the first joint of the robot was placed.

On average, the error on the OX axis was below 1 cm, but there were some spikes in the error signal. The high amplitude errors were due to the sudden change in the reference speed, which was used in controlling the angular velocity through the torque of the joint’s motor. The high amplitude errors were found at the points where the reference changed its path slope and control type. The error had a more continuous shape when the kinematic control was in place, and in the case of the dynamic control, the error tended to oscillate.

**Figure 19:**
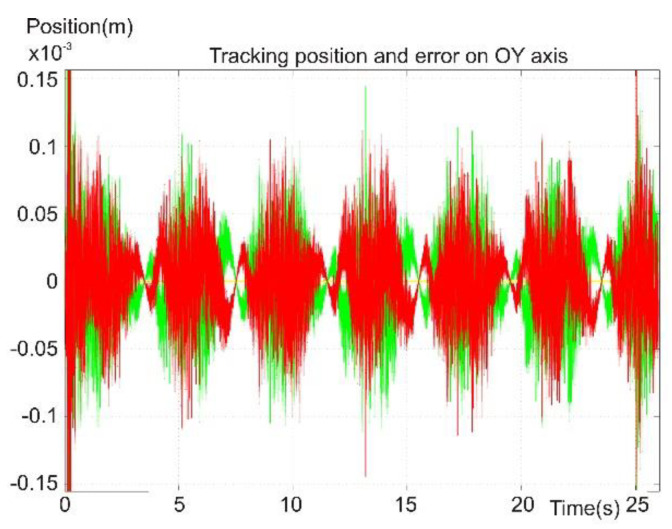
Foot tracking position on the OY axis.

Figure 19 presents the robot foot’s reference, positioning, and error signals on the OY axis. The reference value was zero, and the positioning error was less than 1 mm. On the other two axes, spikes were found in the error signal at the moment when the control law changed.

**Figure 20:**
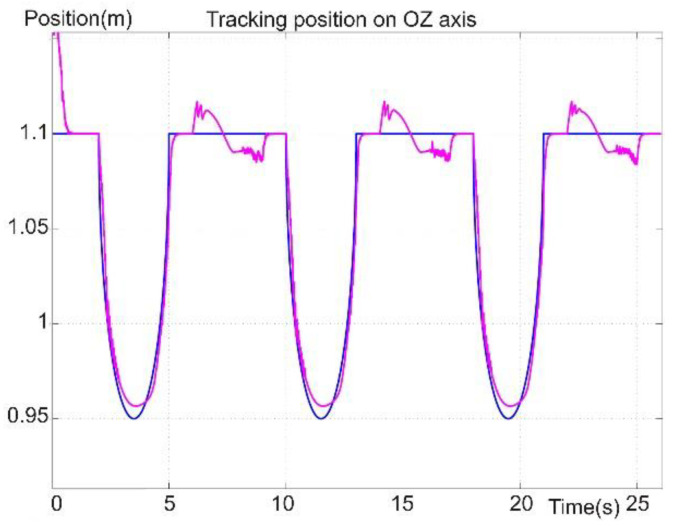
Foot reference and position tracking on the OZ axis.

Figure 20 presents the diagram for the reference and positioning signals of the robot foot on the OZ axis, which corresponds to the perpendicular axis on the support surface, meaning the vertical motion. The diagram presents the foot position during the leg’s swing phase. The leg was positioned on the vertical axis so it would not hit an obstacle or the support surface. Also, the vertical trajectory of the foot was in the support phase, for which the reference was zero. The foot followed a continuous and uniform reference value during the swing phase. In the support and moving-forward phase, a positioning error was observed. The error may have been due to the platform weight compensating at the moment the robot foot crossed the point of intersection with the platform center of the vertical gravity axis. The positioning error became zero on the OZ axis.

**Figure 21:**
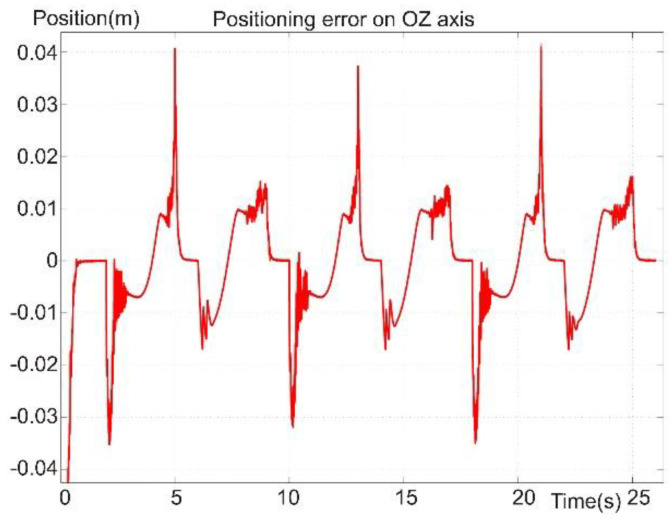
Foot positioning error on the OZ axis.

Figure 17, Figure 18, Figure 19, Figure 20 and Figure 21 present, in the same time frame, the motion of the robot leg stepping three times to move the robot forward. The diagrams show all motion stages for the robot foot to complete a step. They present reference and tracking signals. The homing occurred in the first second of the simulation, and a high error was observed. In the time interval of [2–5 s], the leg moved on the vertical axis and forward, controlled by the kinematic control law to reach a new position for the foot. In the next second of the virtual experiment at [5–6 s], the control method reached the vertical reference position to allow the robot leg to support the robot’s weight. Between 6 and 9 s, the leg moved backward in relation to the robot platform and was controlled by the dynamic SMC method. A different error pattern is observed in Figure 18 and Figure 21, considering that the robot leg supported the weight at this stage. After this stage was completed, the next step continued in the same manner, excluding the homing sequence.

In Figure 21, a maximum error of 4 cm was found at the amplitude peaks and an average value of 1 cm. The high amplitude values appeared as described above when the control laws were switched, which is the subject of future work to stabilize the system at the switch. The error peaks were also due to the sudden shape-change of the reference signal. By changing from a curve signal to a straight line, the derivative part of the controller received an extremely high value, which in turn affected the control signal. The influence should be attenuated or removed entirely in future work.

## 8. Conclusions

**Figure 22:**
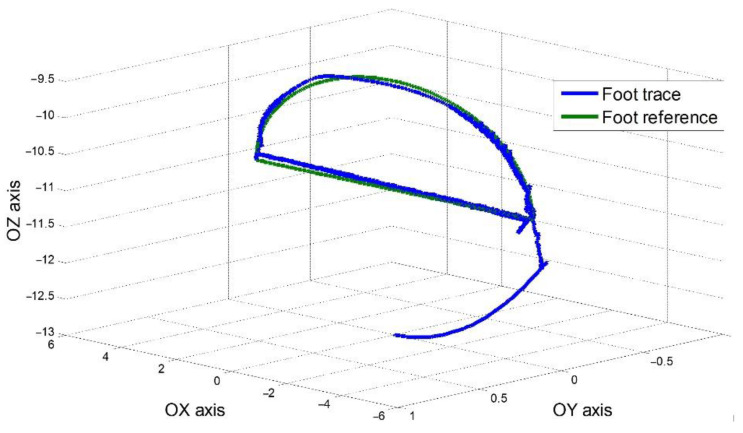
Foot trace in Cartesian coordinates.

To summarize and conclude the results, Figure 22 presents the robot foot trace in a 3D space with the reference pattern. All three steps overlap in the same diagram. The coordinates are given in the Cartesian space. The first stage that was easily found was the homing curve, seen in the lower section of the image. The maximum error was found at the start or end of a step, where the reference system must be improved to avoid sharp changes of direction. For the simulation, a fixed Cartesian coordinates system was considered, with the origin placed in the first joint where the robot top platform joined with to the robot leg. The shape of the horizontal motion was not uniform. The trajectory had minor errors in the range of millimeters and hundreds of micrometers when the control method was not changed, and we considered the shape of the foot trajectory as close to the reference.

Overall, as presented, the dynamic controller was better at following a continuous reference than a simple positioning kinematic controller. Although the positioning was more precise when a dynamic-based controller was used, sudden changes were added in the reference value when changing from a linear trajectory to a half ellipse. Since these were the points where the decision algorithm should also switch the used control method, these points of interest became essential areas of disturbance in the system. We will dedicate our attention to mitigating the reference and switching effects in the reference-tracking algorithm in future work.

The dynamic controller tended to be slower than the kinematic controller in compensating for the disturbance, but it did not override the PI kinematic controller. Moreover, the PI kinematic controller oscillated around the reference value when the robot leg was subjected to exterior forces. The gravitational acceleration acted upon the entire leg when high gains were used inside the control loops to lower the reference tracking time. It resulted in a high tracking error on the vertical axis during the kinematic control. The reference tracking time was considered the time difference between the change to the kinematic control method and until the foot reached the target point, with an error small enough to consider that the position was reached. The target position was chosen near the support surface but far enough for the leg to lower its speed before hitting the surface.

A PI kinematic-based controller was used during the swing motion of the robot leg because high precision was not required to move the robot foot. Instead, a constant foot speed was needed to reach the point of contact with the support surface in a short time. In addition, along with the positioning precision, the controller did not need to compensate for the gravity and inertial forces during the leg and robot motion and could have severe and undesired consequences in the support phase. Therefore, we concluded that a dynamic-based controller was required to compensate for all the inertial forces and to better track the reference.

Finally, the proposed hybrid control efficiently used the two control methods for the mobile walking robot leg. The biggest problem was found during the transition between the two control techniques. 

The main conclusion of the paper was on the decision algorithm side. By having a two-stage decision, the information that could be analyzed offline between simulations defined the outline of the critical decision in each case or phase of the robot. The final algorithm distinguished between robot motion phases and rejected contradictory conditions at the online stage. 

The consequence of the presented hybrid force/position control with a two-stage decision algorithm was that it can successfully be used and further developed for other types of robots or tasks.

Our future work will focus on studies for removing the high peaks of positioning error. At the same time, new, improved simulations are required to visualize the leg motion cycle.

## Figures and Tables

**Table 1:** Robot values.

Dimension	Value
*r*_1_	0.05 [m]
*l*_1_	0.1 [m]
*m*_1_	0.25 [kg]
*r*_2_	0.25 [m]
*l*_2_	0.5 [m]
*m*_2_	1.25 [kg]
*r*_3_	0.35 [m]
*l*_3_	0.7 [m]
*m*_3_	1.76 [kg]

*l_i_* and *r_i_* represent, respectively, leg segment dimensions and the distance from the joint axis to the center of mass for each leg segment, and *m_i_* is the segment’s mass.

**Table 2:** Observer experimental data.

*θ*	Force Sensor m_1_ (*θ*)	Proximity Sensor m_2_ (*θ*)
*θ_D_* *	0.75	0.65
*θ_C_* **	0.2	0.3
*θ_D_*∪*θ_C_* ***	0.05	0.05

* *θ_D_* = dynamic control; ** *θ_C_* = kinematic control; *** *θ_D_∪θ_C_* is the indeterminate area.

**Table 3:** The experimental data after using Equation (6).

*C* = *A*∩*B*	*m* (C)	
*φ*	0	-
*θ_D_*	0.5575	Truth value for *θ_D_* and falsity value for *θ_C_*
*θ_C_*	0.085	Truth value for *θ_C_* and falsity value for *θ_D_*
*θ_D_*∪*θ_C_*	0.0025	Uncertainty between *θ_C_* and *θ_D_*
*θ_D_*∩*θ_C_*	0.355	The contradiction between *θ_C_* and *θ_D_*

**Table 4:** Control probability.

*C* = *A*∩*B*	*A* (Force Sensor)	*B* (Proximity Sensor)	*m* (*C*)	Control Type
*φ*	*φ*	*φ*	0	Robot stopped
*θ_D_*	*θ_D_*	*θ_D_*∪*θ_C_*	0.5575	Dynamic Control
	*θ_D_*∪*θ_C_*	*θ_D_*		
	*θ_D_*	*θ_D_*		
*θ_C_*	*θ_C_*	*θ_D_*∪*θ_C_*	0.085	Cinematic Control
	*θ_D_*∪*θ_C_*	*θ_C_*		
	*θ_C_*	*θ_C_*		
*θ_D_*∪*θ_C_*	*θ_D_*∪*θ_C_*	*θ_D_*∪*θ_C_*	0.0025	Uncertainty
*θ_D_*∩*θ_C_*	*θ_D_*	*θ_C_*	0.355	Contradiction
	*θ_C_*	*θ_D_*		

**Table 5:** The output fuzzy gain computation.

*s*		*NB*	*NM*	*NS*	*Z*	*PS*	*PM*	*PB*
$\dot{s}$		S < −2	−2 < =S < −1	−1 < =S < 0	S = 0	0 < S< = 1	1 < S< = 2	2 < S
*NB*	$\dot{s}<-10$	S	VS	S	M	B	VB	VB
*N*	$-10\leq \dot{s}<0$	M	S	VS	S	M	B	VB
*Z*	$\dot{s}=0$	B	M	S	VS	S	M	B
*PS*	$0<\dot{s}\leq 10$	VB	B	M	S	VS	S	M
*PB*	$10<\dot{s}$	VB	VB	B	M	S	VS	S

## Data Availability

Not applicable.

## Glossary

*θ_i_*	robot leg join rotation
*θ_ref_*	robot joint angular position reference
*θ_real_*	robot joint angular position measured by angular sensors
Δ*θ_i_*	leg joint positioning error
*ω_i_*	joint angular speed
Δ*ω_I_*	joint angular speed error
*r_i_*	distance from the previous joint axis to the *i*th leg segment center of mass
*l_i_*	length of the *i*th leg segment
*m_i_*	mass of the *i*th leg segment
*P_ref_*	position reference of the robot foot
*P_real_*	real position of the robot foot
*J*	Jacobian matrix
*J_ij_*	Jacobian matrix element with coordinates *i* (row) and *j* (column)
*F_sim_*	simulated ground reaction force
*F_real_*	real force given by force sensors placed on robot foot
*F_ground_*	ground reaction force
*d*	distance from robot foot to the ground given by proximity sensors
*s_i_*	*sin*(*θ_i_*)
*c_i_*	*cos*(*θ_i_*)
*s_ij_*	*sin*(*θ_i_* + *θ_j_*)
*c_ij_*	*cos*(*θ_i_* + *θ_j_*)
*τ_P_*	joint torque computed by position control
*τ_F_*	joint torque computed by force control
*τ*	joint torque selected by the decision algorithm
*q_i_*	leg joint used by the dynamic control method
*s*	sliding surface error in terms of SMC
*SMC*	sliding motion control
*O_m_*	extension theory object
*c_m_*	extension theory characteristic
*v_m_*	extension theory measure
*m* (*C*)	the neutrosophic generalized basic belief assignment
*D^θ^*	a hyperpower set
*θ_C_*	neutrosophic kinematic state
*θ_D_*	neutrosophic dynamic state
*θ_D_*∪*θ_C_*	neutrosophic uncertain state
*θ_D_*∩*θ_C_*	neutrosophic contradiction state
